# Can serum metabolic signatures inform on the relationship between healthy lifestyle and colon cancer risk?

**DOI:** 10.1186/s40170-025-00388-0

**Published:** 2025-06-16

**Authors:** Komodo Matta, Vivian Viallon, Anastasia Chrysovalantou Chatziioannou, Nivonirina Robinot, Roland Wedekind, Christina C. Dahm, Agnetha Linn Rostgaard-Hansen, Anne Tjønneland, Therese Truong, Chloé Marques, Pauline Frenoy, Rudolf Kaaks, Renée Turzanski Fortner, Matthias B. Schulze, Sabrina Sieri, Mario Fordellone, Rosario Tumino, Fulvio Ricceri, Tonje Braaten, Therese Haugdahl Nøst, Maria-Jose Sánchez, Olatz Mokoroa-Carollo, Sandra Colorado-Yohar, Camino Trobajo-Sanmartín, Keren Papier, Rhea Harewood, Kostas Tsilidis, Salvatore Vaccarella, Mattias Johansson, Elisabete Weiderpass, Cyrille Delpierre, Sebastien Lamy, Kristin Benjaminsen Borch, Pekka Keski-Rahkonen, Elio Riboli, Heinz Freisling, Marc Gunter, Pietro Ferrari

**Affiliations:** 1 International Agency for Research On Cancer (IARC/WHO), Lyon, France; 2 Department of Public Health, Aarhus University, Bartholins Alle 2, Aarhus C, 8000 Denmark; 3 The Danish Cancer Institute, Strandboulevarden 49, Copenhagen, 2100 Denmark; 4 Department of Public Health, University of Copenhagen, Copenhagen, Denmark; 5 Paris-Saclay University, UVSQ, Gustave Roussy, CESP, Inserm Villejuif, France; 6 Division of Cancer Epidemiology, German Cancer Research Center (DKFZ), Heidelberg, Germany; 7 Department of Research, Cancer Registry of Norway, Norwegian Institute of Public Health, Oslo, Norway; 8 Department of Molecular Epidemiology, German Institute of Human Nutrition Potsdam-Rehbruecke, Nuthetal, Germany; 9 Institute of Nutritional Science, University of Potsdam, Nuthetal, Germany; 10 Epidemiology and Prevention Unit, Fondazione IRCCS Istituto Nazionale Dei Tumori Di Milano Via Venezian, Milan, 20133 Italy; 11 Unit of Medical Statistics, University of Naples “Vanvitelli”, Naples, Italy; 12 Hyblean Association For Epidemiology Research, AIRE-ONLUS, Ragusa, Italy; 13 Department of Clinical and Biological Sciences, Centre for Biostatistics, Epidemiology, and Public Health (C-BEPH), University of Turin, Turin, Italy; 14 Department of Community Medicine, Faculty of Health Sciences, UiT The Arctic University of Norway, Tromsø, Norway; 15 Faculty of Nursing and Health Sciences, Nord University, Bodø, Norway; 16 Department of Public Health and Nursing, HUNT Research Center, NTNU - Norwegian University of Science and Technology, Trondheim, Norway; 17 Levanger Hospital, Nord-Trøndelag Hospital Trust, Levanger, Norway; 18 Escuela Andaluza de Salud Pública (EASP), Granada, 18011 Spain; 19 Instituto de Investigación Biosanitaria Ibs.GRANADA, Granada, 18012 Spain; 20 Centro de Investigación Biomédica en Red de Epidemiología y Salud Pública (CIBERESP), Madrid, 28029 Spain; 21 Sub Directorate for Public Health and Addictions of Gipuzkoa, Ministry of Health of the Basque Government, San Sebastian, Spain; 22 Epidemiology of Chronic and Communicable Diseases Group, BioGipuzkoa (BioDonostia) Health Research Institute, San Sebastián, Spain; 23 Department of Epidemiology, Murcia Regional Health Council, IMIB-Arrixaca, Murcia, Spain; 24 Research Group On Demography and Health, National Faculty of Public Health, University of Antioquia, Medellín, Colombia; 25 Instituto de Salud Pública y Laboral de Navarra, Pamplona, 31003 Spain; 26 Navarre Institute for Health Research (IdiSNA), Pamplona, 31008 Spain; 27 Department of Population Health, Cancer Epidemiology Unit, Nuffield, University of Oxford, Oxford, UK; 28 Department of Surgery and Cancer, Faculty of Medicine, Imperial College London, London, UK; 29 Department of Epidemiology and Biostatistics, School of Public Health, Faculty of Medicine, Imperial College London, London, UK; 30 Department of Hygiene and Epidemiology, University of Ioannina School of Medicine, Ioannina, Greece; 31 Center for Epidemiology and Research in POPulation Health (CERPOP), Inserm, Université de Toulouse III, Toulouse, France; 32 Tarn Cancer Registry, Oncopole Claudius Regaud, Toulouse, France

**Keywords:** Colon cancer, Healthy Lifestyle, Untargeted metabolomics, Socioeconomic position, Sex, LASSO regression

## Abstract

**Background:**

Colon cancer is strongly influenced by lifestyle factors. Sociodemographic factors like sex and socioeconomic position (SEP) might modulate the relationship between lifestyle and colon cancer risk. Metabolomics offers potential to uncover biological mechanisms linking lifestyle and colon cancer.

**Methods:**

Lifestyle and untargeted metabolomic data were available from a nested case–control study within the European Prospective Investigation into Cancer and Nutrition (EPIC), including 1,067 colon cancer cases and 1,067 controls matched on age, sex, study centre, and blood collection time. Serum samples were analyzed using liquid chromatography-mass spectrometry. The Healthy Lifestyle Index (HLI) score was derived from smoking habits, alcohol intake, body mass index (BMI), physical activity, and diet. Penalised regression was applied in controls to derive metabolic signatures for the HLI and the lifestyle components. Associations of lifestyle factors and the metabolic signatures with colon cancer risk were estimated in conditional logistic regression models, overall and by sex and SEP.

**Results:**

The HLI score was inversely associated with colon cancer risk, with an odds ratio (OR) per 1-standard deviation (SD) increment equal to 0.79; 95% CI: 0.71, 0.87. The metabolic signature of HLI, comprising 130 features, was moderately correlated with HLI (r = 0.59; 94% CI: 0.56, 0.61), and was inversely associated with colon cancer risk (OR = 0.86; 95% CI: 0.78, 0.95). After adjustment for the HLI score, the association of the metabolic signature of HLI and colon cancer risk was null (OR = 1.00, 95% CI 0.88, 1.13). Associations of lifestyle factors and the metabolic signature with colon cancer risk were consistently stronger for men than for women and did not differ by SEP.

**Conclusions:**

In this study across seven European countries, healthy lifestyle was inversely associated with colon cancer risk, with stronger associations in men than women and no differences across SEP. However, the serum metabolic signatures after adjustment for lifestyle factors were not found to be associated with colon cancer risk, suggesting that lifestyle impacts colon cancer through mechanisms not captured by the signatures.

**Supplementary Information:**

The online version contains supplementary material available at 10.1186/s40170-025-00388-0.

## Background

Colon cancer stands as a prominent global health concern, ranking fourth most common cancer in incidence and fifth in mortality worldwide [1]. The heterogeneity of colon cancer incidence rates across geographical regions suggests the involvement of lifestyle factors in its aetiology [1, 2]. A recent systematic review by the World Cancer Research Fund/American Institute for Cancer Research (WCRF/AICR) reported strong evidence linking lifestyle factors, such as physical inactivity, body fatness, and poor dietary habits to increased risk of developing colorectal cancers [3], and making improvements in lifestyle behaviours has been linked to decreased risk [4].

Lifestyle patterns and their impacts on health outcomes are also influenced by sociodemographic factors such as sex and socioeconomic position (SEP) [4–8]. Women generally engage in healthier lifestyle behaviours than men [7, 9], while individuals with lower SEP are more likely to adopt riskier behaviours compared to those with higher SEP [10–12]. Additionally, sex disparities have been observed in the association between lifestyle and colon cancer risk, especially by anatomical subsite, with men more likely to develop distal colon cancer, and women at higher risk for proximal colon cancer [7, 13–15]. SEP may influence biological functioning through differential environmental, behavioural, and psychosocial exposures that may influence stress responses [16]. While lower SEP has consistently been associated with higher cancer mortality across nearly all cancer types [17], its specific link to colon cancer incidence remains inconclusive [12]. The modulating role of sex and SEP in relationship between lifestyle and colon cancer risk calls for further exploration.

While the influence of lifestyle on colon cancer is well-documented, the underlying biological mechanisms remain poorly understood. It is possible that lifestyle factors may induce metabolic perturbations, which, in turn, contribute to cancer development. Metabolomics, the large-scale study of small molecules in biological samples such as blood, has emerged as a powerful tool to explore these metabolic pathways. Prior research within the European Prospective Investigation into Cancer and Nutrition (EPIC) study has identified metabolic biomarkers associated with alcohol consumption and pancreatic and liver cancer risk [18], healthy lifestyle and hepatocellular carcinoma [19], and body size and colorectal cancer [20], which were more strongly associated with the outcome than the exposure itself. These studies suggest that metabolomics may hold potential for better understanding the underlying biological pathways in the relationship of lifestyle and colon cancer development.

In this study, we analysed the associations between colon cancer risk and lifestyle factors, measured as the Healthy Lifestyle Index (HLI), using data from a nested case–control study in the EPIC cohort. Additionally, we leveraged untargeted metabolomics data to derive metabolic signatures of the HLI and its components. We evaluated how sex and SEP modulate the relationships between lifestyle, metabolomic profiles, and colon cancer risk.

## Materials and methods

### Study population

EPIC is a large prospective cohort, which enrolled 521,323 adults aged 35 to 70 from 23 centres in 10 European countries (Denmark, France, Germany, Greece, Italy, the Netherlands, Norway, Spain, Sweden, and the UK) between 1991 and 2000 to study the associations of diet, lifestyle, environmental and metabolic factors with cancer and other diseases [21]. All study participants provided informed consent for the use of their lifestyle and metabolic data, and ethical approval was obtained from the participating centres and ethics committees.

### Lifestyle data

Upon enrolment, participants completed a lifestyle questionnaire, providing information on lifestyle habits including tobacco smoking, alcohol consumption, physical activity, exogenous hormone use, medical and reproductive history, as well as highest education level attained [21]. Education level was used as a proxy for socioeconomic position (SEP) and was analysed as a dichotomous variable: low SEP comprising no schooling, primary, or technical school completed, and high SEP comprising secondary school or higher, including university degrees. Dietary data was collected through validated country-specific questionnaires [22–25] and later harmonised with the food composition databased (EPIC Nutrient Database, ENDB) [22]. Anthropometric measurements were taken by trained study personnel or by self-reported data which has been validated [26]. Details of the lifestyle questionnaires have been described elsewhere [21, 22, 24, 27–29].

### Metabolomics data

Biological samples of approximately 80% of EPIC participants were collected among cancer-free study participants upon recruitment and stored at IARC (Lyon, France) in − 196 °C liquid nitrogen, or locally (Denmark, − 150 °C nitrogen vapor; Sweden, − 80 °C freezers). Serum untargeted metabolomics data were acquired at IARC using a UHPLC-QTOF-MS system (1290 Binary Liquid Chromatography system, 6550 Quadrupole Time-of-Flight mass spectrometer; Agilent Technologies, Santa Clara, CA). Reversed phase chromatography and electrospray ionisation were employed in both positive and negative polarities. Samples were analysed in five analytical batches, each consisting of five 96-well plates. Samples were randomised across the batches, with the case–control pairs analysed next to each other.

For the pre-processing of the raw data Agilent MassHunter Profinder 10.0.2.162, and Mass Profiler Professional 14.9.1 (MPP) software were used, applying Agilent’s recursive feature finding procedure for each analytical batch. A combined target list was generated from all batches and a targeted feature extraction from all the study samples was performed against the list of targets.

To enable quality control (QC) and background exclusion, pooled QC samples and blank samples were included in the analysis. Details of sample preparation and analysis as well as raw data pre-processing are available in the Supplementary Methods.

### Nested case–control study

This analysis used data from a case–control study nested within the EPIC cohort, which included 2,242 participants (1,121 incident colon cancer cases and 1,121 risk-matched controls) with available biological samples. Incident cancer cases were identified from health insurance records, cancer and pathology registries, and active follow-up. Cases were defined using the International Classification of Disease, 10th revision (ICD-10), and the International Diseases for Oncology, 2nd revision (ICD-O-2). Controls were risk-set matched 1:1 by age at recruitment (± 6 months), sex, study centre, time and fasting status of blood collection, follow-up time since blood collection, and, for women, menopausal status and/or phrase of menstrual cycle at blood collection.

### Statistical analysis

#### Healthy lifestyle index

As lifestyle behaviours are interrelated and rarely occur in isolation, a composite score, i.e. Healthy Lifestyle Index (HLI), was derived to assess the cumulative impact of lifestyle on disease risk in addition to assessing each lifestyle factor independently [30]. Previous iterations of HLI have been simple composite scores in which each lifestyle component is given equal weight; however, this can produce biased estimates of disease risk, as it assumes all components have the same relationship with the disease [31]. A weighted, outcome-specific approach was employed.

The HLI score was calculated as an outcome-specific weighted sum of the five lifestyle components, split into five exposure level categories: smoking (never, ex-smokers quit > 10 years, ex-smokers quit ≤ 10 years, current smokers ≤ 15 cigarettes/day, and current smokers > 15 cigarettes/day), alcohol consumption at recruitment (< 6, 6–12, 12–24, 24–60, and ≥ 60 g/day), body mass index (BMI) (< 22, 22–24, 24–26, 26–30, and ≥ 30 kg/m^2^), physical activity (Metabolic Equivalent of Task [MET] hours/week quintiles), and dietary habits (modified relative mediterranean diet score [mrMDS] quintiles) [32], where a higher score corresponded to a greater propensity to engage in healthy lifestyle behaviours. Data-driven weights for each category of each lifestyle component, scaled for unit variance, were derived from the parameters of the main effects $\left({w}_{k}\right)$ in outcome-specific Cox models on the whole EPIC study population, adjusted for education level and height, and stratified by study centre, recruitment age, and sex, with the reference category having a weight of zero. The HLI was thus defined as ${\sum }_{k=1}^{5} {w}_{k}*{\left(Lifestyle component\right)}_{k}$ where ${w}_{k}$ is the weight of each category of the lifestyle component. The final HLI score was then centred and scaled for unit variance by the control distribution in the nested case–control study population. Methods of the HLI derivation are explained in more detail elsewhere [31].

#### Metabolic signatures

Metabolomics data were sequentially filtered and transformed following a pipeline summarised in Figure S1 and described in detail elsewhere [33]. In brief, features were only included if feature intensities were a minimum ratio of fivefold over the blank samples. Filtering of features was done if features were missing across 50% of samples within at least one plate. Samples were filtered if > 50% of features were missing for the sample and if identified as strong analytical outliers in a principal components analysis. Missing values were imputed across plates with k-nearest neighbour (KNN) with $k=10$. Features were normalised relative to the QC samples using the systematic error removal using random forest (SERRF) method [34]and subsequently log-transformed and scaled. Samples were additionally excluded if they did not have anthropometric and lifestyle data, if they lacked data in both positive and negative modes or were missing the matched case–control pair.

The main sources of variation among covariates and potential confounders (i.e. country, study centre, education, age, height, batch, plate, HLI) in the metabolomics data were identified using Principal Component Partial R-square (PC-PR2), a multivariate technique that combines principal component analysis and multiple linear regression [35]. To account for and remove remaining unwanted technical variability (e.g., batch effects and study centre) while preserving the biological variability of the metabolomics data, linear mixed-effect models were applied. Residuals from these models were extracted and used in subsequent analyses.

Metabolic signatures were derived using the control population. A nested tenfold cross validation was used to assess different dimension reduction methods for deriving the metabolic signature (Table S1), with minimising root mean squared error (RMSE) and maximising correlation of the signature with HLI (continuous) as model performance indicators. Based on model performance, least absolute shrinkage and selection operator (LASSO) regression was used to derive the metabolic signatures of the HLI and the lifestyle components in subsequent analyses. LASSO is a penalised regression that uses a penalty term (λ) to reduce dimensionality, which can address the redundancy of fractionated features corresponding to the same metabolite, as well as the redundancy of features present in both positive mode and negative mode [36, 37].

#### Assessment of HLI and metabolic signatures

After the derivation of the metabolic signatures of HLI and its components, as well as of HLI by sex and SEP (i.e. “subgroup-specific”), Pearson correlation was used to assess the correlation between metabolic signatures to the HLI, with and without adjustment for sex and SEP. Subgroup-specific metabolic signatures of HLI were derived by first subsetting the data by sex and SEP and then running a separate LASSO model for each subgroup. Subgroup-specific metabolic signatures of HLI were then compared with the global metabolic signature of HLI by each subgroup, using partial Pearson correlations adjusted for sex and SEP, and the subgroup-specific metabolite signatures were not found to be different from the global metabolic signature of HLI; thus the global metabolic signature was used for subsequent analyses (Table S2).

For component-specific metabolic signatures, the smoking signature was derived from an ordinal score for smoking status (i.e. 0 = never, 1 = ex-smokers quit > 10 years, 2 = ex-smokers quit <  = 10 years, 3 = current smokers <  = 15 cigarettes/day, 4 = current smokers > 15 cigarettes/day), the alcohol signature was derived from the continuous variable for alcohol consumption [g/day], the BMI signature was derived from the continuous variable for BMI [kg/m^2^], the physical activity signature was derived from the continuous variable for weekly METs, and the diet signature was derived from the continuous variable for modified Mediterranean score, which has been described elsewhere [32]. All lifestyle components and their component-specific metabolic signature derivatives were centred and scaled to unit variance by the control population.

#### Logistic regression analyses

The associations between lifestyle variables (HLI and the five lifestyle components) and their metabolic signatures and colon cancer risk were evaluated using conditional logistic regression models, adjusted for education and height, and stratified by the matched case set. Odds ratios (ORs) and 95% confidence intervals (CIs) reported reflect a one standard deviation (sd) increase. All statistical tests were two-sided and *p*-values < 0.05 were considered statistically significant. The main models included as exposures: HLI unadjusted for metabolic signature, the metabolic signature of HLI unadjusted for HLI, and a mutually adjusted model; and as outcomes: colon cancer overall, and distal and proximal colon subsites. Estimates by sex and by SEP were modelled by including an interaction term between the dichotomous modulating variable (for sex, male = 0, female = 1; for SEP, low SEP = 0, high SEP = 1) and the exposure variables. For the component-specific models, unadjusted estimates were modelled with either the lifestyle component or the metabolic signature of the lifestyle component; adjusted estimates were modelled with the metabolic signature of each respective lifestyle component, adjusted for all five lifestyle components and covariates.

All statistical analyses were performed in R software version 4.1.2 (R Foundation for Statistical Computing).

## Results

### Participant characteristics

The study population is described in Table 1. 1,067 incident colon cancer cases and their 1:1 risk-matched controls, with metabolomic data available in both positive and negative ion modes, were included in this study. Cases and controls were similar with respect to age at blood collection, height, alcohol consumption, diet, smoking status, and education level. Compared to controls, cases had higher BMI on average and lower physical activity. On average, the HLI scores were lower and more variable for cases compared to controls. For the derivation of the HLI score, lifestyle component weights for each level of exposure are listed in Table S3. Participant characteristics by sex and by SEP are available in Table S4. Men had on average lower and more variable HLI scores than women. Men tended to have higher BMIs, smoke more, and drink more alcohol, while women were more physically active.

**Table 1:** Characteristics of the Colon Cancer Nested Case–Control study^1^ participants in EPIC. Unless otherwise indicated, medians (interquartile range [IQR]) are presented for continuous variables and frequencies (%) for categorical variables

Characteristic	Controls (*N* = 1,067)	Cases (*N *= 1,067)
**Sex**		
Male	473 (44.3%)	473 (44.3%)
Female	594 (55.7%)	594 (55.7%)
**Age at blood collection [years]**	57.4 (51.4, 61.9)	57.5 (51.6, 62.0)
**Height [cm]**	164.6 (158, 171)	165.2 (159, 172)
**Weight [kg]**	71.3 (62.9, 80.0)	73.0 (64.4, 84.6)
**Body Mass Index [kg/m**^**2**^**]**	26.0 (23.6, 28.8)	26.9 (24.1, 29.7)
**Alcohol at recruitment [g/d]**	8.1 (0.8, 22.7)	7.1 (0.7, 24.3)
**Physical Activity [MET hours/week]**	79.8 (46.3, 122.9)	74.1 (42.0, 118.1)
**Modified-relative Mediterranean Diet Score (mrMDS)**	27 (23, 32)	27 (23, 32)
**Smoking Status**		
Current, > 15 cigarettes/day	86 (8.1%)	97 (9.1%)
Current, ≤ 15 cigarettes/day	218 (20.4%)	206 (19.3%)
Former, quit ≤ 10 years	95 (8.9%)	126 (11.8%)
Former, quit > 10 years	215 (20.1%)	212 (19.9%)
Never	453 (42.5%)	426 (39.9%)
**Highest Education**		
None	92 (8.7%)	104 (10.0%)
Primary	396 (37.5%)	348 (33.4%)
Technical/professional	219 (20.5%)	232 (22.2%)
Secondary	155 (14.5%)	176 (16.9%)
University or higher	171 (16.0%)	159 (15.2%)
Not specified	34 (3.2%)	48 (4.4%)
**Country**		
France	41 (3.8%)	41 (3.8%)
Italy	283 (26.5%)	283 (26.5%)
Spain	240 (22.5%)	240 (22.5%)
United Kingdom	161 (15.1%)	161 (15.1%)
Netherlands	100 (9.4%)	100 (9.4%)
Germany	106 (9.9%)	106 (9.9%)
Denmark	136 (12.7%)	136 (12.7%)
**Tumour site**		
Proximal colon	-	486 (45.5%)
Distal colon	-	510 (47.8%
Colon, unspecified	-	71 (6.7%)
**Healthy Lifestyle Index (HLI)**^2^	0 (SD = 1)	−0.19 (SD = 1.05)
**Metabolic Signature of HLI (MetSig HLI)**^2^	0 (SD = 1)	−0.11 (SD = 1.07)

*Abbreviations*: *MET* Metabolic Equivalent of Task

^1^Matching factors were age, sex, study centre, fasting status, and follow-up time since blood collection. Women were additionally matched for menopausal status and phase of menstrual cycle at blood collection

^2^Presented as mean (standard deviation [SD])

## Metabolic signature of HLI

After preprocessing and filtering of untargeted metabolomic data, 2,083 metabolic features (930 in positive ion mode and 1,153 is negative ion mode) were retained for analysis. Metabolic signatures of the HLI score and of its components were derived from the control population. Of the 2,083 features, the LASSO-derived metabolic signature of HLI (MetSig HLI) comprised 130 features (61 positive, 69 negative). The most contributing features are described in Table 2 and Figure S2. The MetSig HLI was strongly correlated with the HLI in the controls when unadjusted (Spearman correlation r = 0.67; 95% CI: 0.64, 0.70), and moderately correlated when adjusted for sex and SEP (Spearman correlation r = 0.59; 95% CI: 0.56, 0.61). The correlation was higher among men (r = 0.63; 95% CI: 0.59, 0.67) than among women (r = 0.53; 95% CI: 0.49, 0.57) and did not differ by SEP (Table 3).

**Table 2:** Top fifteen features contributing to metabolic signature of the Healthy Lifestyle Index (HLI) ranked by loading coefficient, including loading coefficients for component-specific metabolic signatures and univariate ORs (95% CI) for colon cancer risk

			**Loading Coefficients**						**Colon Cancer OR (95% CI)**^1^
**Mass (Da, monoisotopic)**	**Retention Time (min)**	**Ion polarity**	**HLI**	**Smoking**	**Alcohol**	**BMI**	**Physical Activity**	**Diet**	
126.0067	0.922018	negative	−0.086	0.043	0.041			0.041	0.94 (0.80, 1.10)
83.0734	0.909614	positive	0.084	−0.029				0.33	0.95 (0.80, 1.12)
230.0752	0.932194	positive	−0.080		4.252				0.94, 0.80, 1.12)
259.8975	0.868995	negative	−0.077	0.219					0.98 (0.83, 1.16)
200.013	3.849583	negative	−0.077	0.171	0.305	−0.048			0.92 (0.78, 1.09)
113.0585	0.648592	positive	−0.070	< 0.001	0.154		4.394		0.96 (0.82, 1.12)
152.0327	1.314172	negative	−0.066					−0.235	1.07 (0.90, 1.26)
450.2613	6.849182	negative	−0.066	0.057	0.941		−4.889		1.04 (0.85, 1.27)
232.0016	3.3877	negative	−0.059	−0.023	−0.814	−0.033	2.424		0.93 (0.79, 1.09)
202.0141	2.746663	positive	−0.055	0.032	0.590				1.06 (0.89, 1.26)
229.003	1.963065	negative	0.050	−0.020					0.88 (0.75, 1.04)
294.0254	0.921684	negative	−0.049	0.011	2.380				1.01 (0.85, 1.20)
198.0379	1.099398	negative	−0.048	0.200					1.08 (0.90, 1.29)
146.0683	0.647574	negative	0.047			−0.183			0.95 (0.81, 1.13)
214.8957	0.900464	negative	0.046	−0.090	−0.016				1.08 (0.91, 1.28)

^1^ Odds ratios for colon cancer risk are adjusted for HLI, height, education, and stratified by the matched case-set. Confidence intervals adjusted for multiple testing using Bonferroni correction

**Table 3:** Partial correlations^1^ (95% CI) of metabolic signature of the Healthy Lifestyle Index (MetSig HLI), overall and by sex and socio-economic position (SEP), and of component-specific metabolic signatures with their respective lifestyle components, the HLI score, and the MetSig HLI

Metabolic Signatures	k_feature_	Partial Correlations^1^ with		
		**Lifestyle component**^**2**^	**HLI score**	**MetSig HLI**
**MetSig HLI**	130	-	0.59 (0.56, 0.61)	1
Male	130	-	0.63 (0.59, 0.67)	1
Female	130	-	0.53 (0.49, 0.57)	1
Low SEP	130	-	0.58 (0.54, 0.61)	1
High SEP	130	-	0.60 (0.55, 0.61)	1
**Component Specific MetSig**				
Smoking	72	0.65 (0.62, 0.69)	−0.27 (−0.31, −0.23)	−0.70 (−0.72, −0.68)
Alcohol	48	0.58 (0.55, 0.60)	−0.30 (−0.34, −0.26)	−0.78(−0.80, −0.76)
BMI	117	0.61 (0.58, 0.64)	−0.31 (−0.35, −0.27)	−0.58 (0.61, −0.55)
Physical Activity	19	0.11 (0.07, 0.15)	< 0.01 (−0.04, 0.04)	0.32 (0.28, 0.36)
Diet	102	0.44 (0.40, 0.47)	0.14 (0.10, 0.18)	0.36 (0.32, 0. 40)

*Abbreviations*: *k*_*feature*_ number of features

^1^ Adjusted for sex and SEP, and stratified by the matched case-set. Component-specific metabolic signatures were mutually adjusted by all five components

^2^ Correlations reported between component-specific metabolic signatures and their respective lifestyle component, e.g. Smoking with MetSig Smoking, Alcohol with MetSig Alcohol, etc.

### Associations with colon cancer

**Fig. 1:**
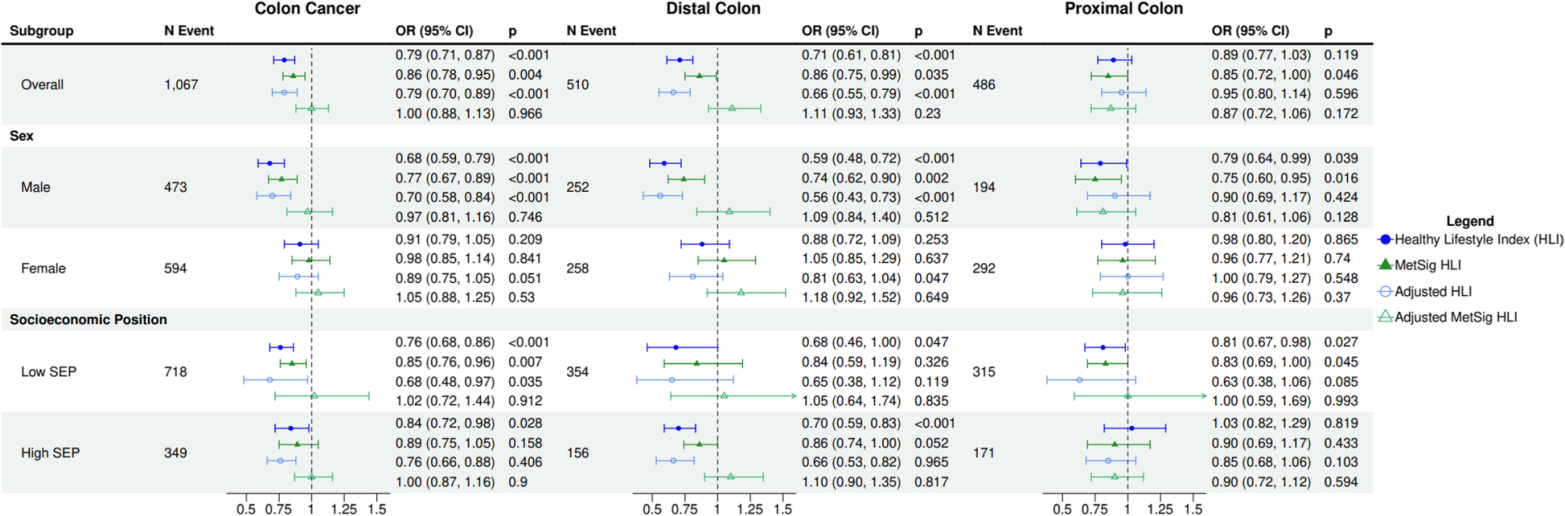
ORs (95% CI) for colon cancer risk and the Healthy Lifestyle Index (HLI) and the metabolic signature of HLI (MetSig HLI), overall, by sex and by SEP. All estimates are reported for a 1-standard deviation increase, adjusted for height and educational level, and stratified by the matched case-set. Unadjusted estimates are modelled with either HLI or MetSig HLI and covariates. Adjusted estimates reflect a mutually adjusted model with both HLI and MetSig HLI, and covariates

The HLI was associated with colon cancer (OR_HLI_ = 0.79; 95% CI 0.71, 0.87), particularly with distal colon cancer (OR_HLI_ = 0.71; 95% CI 0.61, 0.81) (Fig. 1). The association between the HLI and colon cancer was stronger in men than in women (OR_HLI_ = 0.68; 95% CI 0.59, 0.79 in men; OR_HLI_ = 0.91; 95% CI 0.79, 1.05 in women; p-heterogeneity = 0.004) and did not differ significantly by SEP (p-heterogeneity = 0.32). The MetSig HLI was similarly associated with colon cancer, with stronger associations for men than in women (OR_MetSig HLI_ = 0.86; 0.78, 0.95 overall; OR_MetSig HLI_ = 0.77; 0.67, 0.89 in men; OR_MetSig HLI_ = 0.98; 0.85, 1.14 in women). However, when both the HLI score and the MetSig HLI were included in the same model (mutually adjusted) the associations with MetSig HLI became null (OR_MetSig HLI Adjusted_ = 1.00, 0.88, 1.13) while the associations with HLI remained virtually unchanged (OR_HLI Adjusted_ = 0.79, 0.70, 0.89).

## Component-specific metabolic signatures

**Fig. 2:**
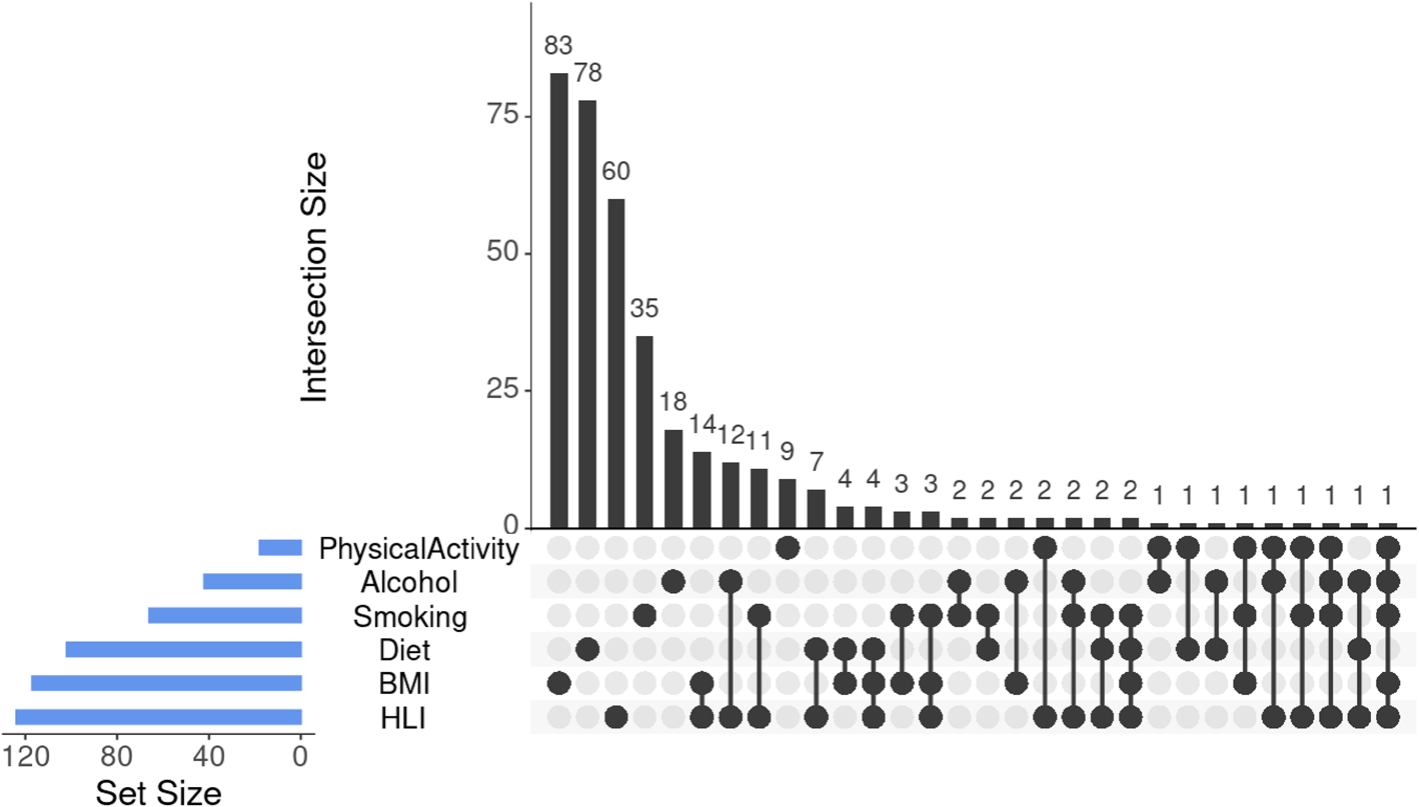
UpSet plot of features selected in the metabolic signatures of the Healthy Lifestyle Index (HLI) and of the lifestyle components and their intersections

Metabolic signatures for each of the lifestyle components were derived using the same methods to derive the MetSig HLI and are described in Table 3. The component-specific metabolic signatures comprised 117 features for BMI, 102 features for diet, 72 features for smoking, 48 features for alcohol,, and 19 features for physical activity. The metabolic signatures for smoking, alcohol consumption, and BMI were inversely correlated with the HLI score and with the metabolic signature of HLI, while the metabolic signatures of physical activity and diet were positively correlated with HLI and its metabolic signature (Table 3). Component-specific metabolic signatures were weakly correlated with the HLI score, but the smoking and alcohol signatures were strongly inversely correlated with the MetSig HLI (r = −0.70; 95% CI: −0.72, −0.68 for smoking; r = −0.78; 95% CI: −0.80, −0.76 for alcohol). The metabolic signatures of smoking and of alcohol shared the greatest proportion of features with the HLI MetSig (40% of features in the MetSig Smoking and 50% of the features in MetSig Alcohol overlapped with the features in MetSig HLI). Features were shared between the MetSig HLI and the metabolic signatures of BMI (14 features), alcohol (12 features), and smoking (11 features) exclusively, while fewer features were shared among multiple metabolic signatures (Fig. 2).

### Associations with colon cancer

**Fig. 3:**
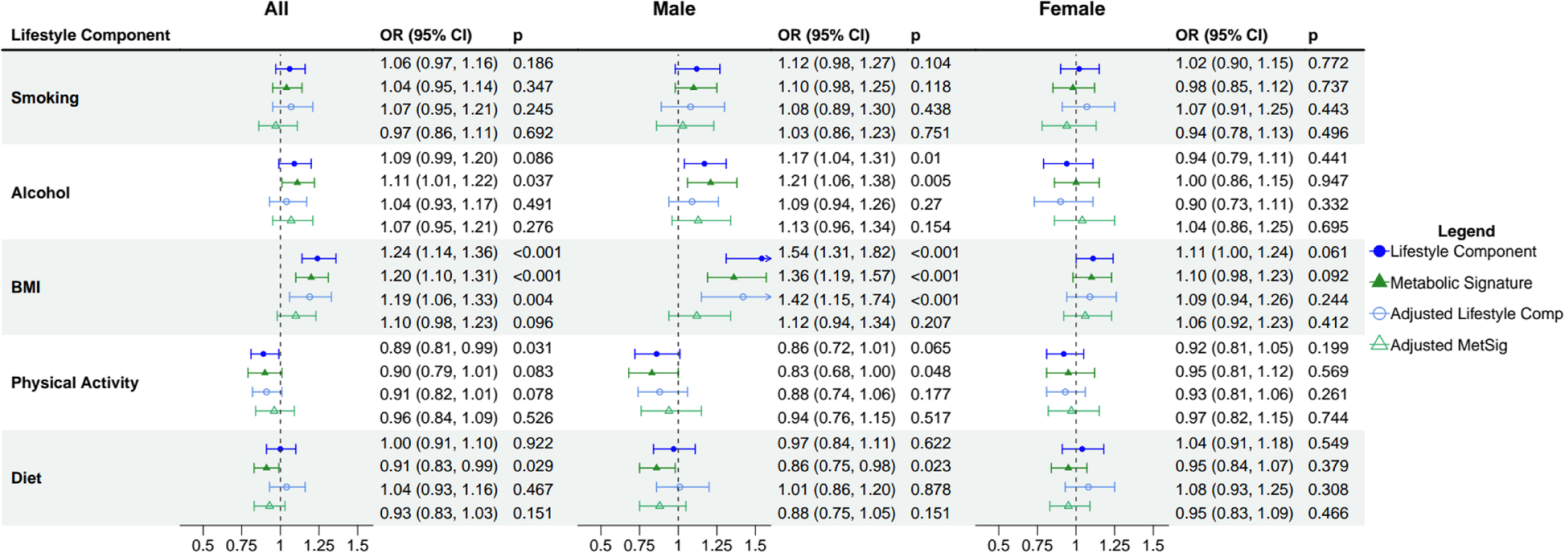
ORs (95% CI) for colon cancer risk and lifestyle components and their metabolic signatures. All estimates are reported for a 1-standard deviation increase and all models were stratified by match case-set. Unadjusted estimates are modelled with either the lifestyle component or the metabolic signature (MetSig). Adjusted estimates are modelled with the metabolic signature of each respective lifestyle component, adjusted for all five lifestyle components and covariates

Lifestyle components and their metabolic signatures were related to colon cancer (Fig. 3). BMI and the metabolic signature of BMI were positively associated with colon cancer in the unadjusted models (OR_BMI_ = 1.24; 95% CI: 1.14, 1.36; OR_MetSig BMI_ = 1.20; 95% CI: 1.10, 1.31). After fully adjusting for all lifestyle components and covariates, BMI remained associated with colon cancer (OR_BMI_ = 1.19; 95% CI: 1.06, 1.33), but the association with MetSig BMI was attenuated (OR_MetSig BMI_ = 1.10; 95% CI: 0.98, 1.23). Interestingly, although diet was not associated with colon cancer (OR_diet_ = 1.00; 95% CI: 0.91, 1.10), its metabolic signature had an inverse association (OR_MetSig diet_ = 0.91, 0.83, 0.99), in the unadjusted models. After full adjustment, the association lost significance (fully adjusted model OR_MetSig diet_ = 0.93, 0.83, 1.03). In men but not in women, alcohol and its metabolic signature were associated with colon cancer in the unadjusted models (Male OR_alcohol_ = 1.17; 95% CI: 1.04, 1.31; Male OR_MetSig alcohol_ = 1.21; 95% CI: 1.06, 1.38). Again, these associations were attenuated in the fully adjusted model. Physical activity was weakly inversely associated with colon cancer (OR = 0.91; 95% CI: 0.82, 1.01) but its metabolic signature was not (OR_MetSig PA_ = 0.96; 95% CI: 0.84, 1.09). No associations were observed for smoking and its metabolic signature with colon cancer. Model adjustments are listed in Table S5.

## Discussion

In this study, we investigated the associations between healthy lifestyle factors and colon cancer risk, integrating untargeted metabolomic data to identify metabolic signatures of the HLI score and its components. Both the HLI score and its metabolic signature (MetSig HLI) were inversely associated with colon cancer risk in separate models. However, after mutual adjustment, only the HLI remained significantly associated with colon cancer, while the associations with the HLI MetSig were null, suggesting that healthy lifestyle behaviours have an impact on colon cancer risk in ways not captured by the metabolic signature.

The association between the HLI and colon cancer risk was stronger in men than in women, consistent with existing evidence [9, 13, 15]. The sex-specific differences in the associations between lifestyle and colon cancer observed in our study may be due to the differential variability of lifestyle exposures across sex, with men engaging in unhealthier habits than women, e.g., consuming more alcohol, smoking more, and having greater rates of obesity. In our study, men had on average lower and more variable HLI scores than women. Also, under the hypothesis that the underlying metabolic processes related to lifestyle were different in men and women, we derived sex-specific metabolic signatures of HLI and found that the MetSig HLI in men had higher correlation with the HLI score than the signatures in women, yet sex-specific signatures did not markedly differ from the overall signature. Our findings are similar to a previous study that derived metabolic signatures reflecting adherence to the recommendations of the World Cancer Research Fund (WCRF)/AICR score using targeted metabolomics in the EPIC cohort [8], which also found a stronger inverse association between healthy lifestyle and colorectal cancer risk in men than in women, and the associations between the metabolic signatures and colorectal cancer were no longer observed after adjustment for the lifestyle score.

We did not find the associations between the HLI and colon cancer risk to differ by socioeconomic position (SEP), despite SEP being a known determinant of lifestyle behaviours, particularly with respect to smoking habits, alcohol drinking, obesity, physical inactivity, and poor diet quality [10, 11]. In our study, participants with lower SEP had on average lower and more variable HLI scores, higher smoking and obesity rates, and poorer dietary habits than participants with higher SEP. However, the associations between lifestyle and colon cancer were similar across SEP groups, and SEP-specific metabolic signatures did not provide additional information beyond the overall HLI signatures.

In our study, we also examined the five lifestyle components of the HLI separately, and found that BMI was the component most strongly associated with colon cancer risk, in line with existing epidemiological evidence [38, 39]. Alcohol consumption was associated with colon cancer risk in men but not in women, consistently with some previous observations [39, 40]. Interestingly, metabolic signature of diet exhibited a stronger association with colon cancer risk than the diet score itself, but not after adjusting for all lifestyle components.

Overall, we did not observe significant associations between metabolic signatures and colon cancer risk, underscoring our gap in knowledge of the mechanisms by which lifestyle factors contribute to the development of colon cancer. Though the metabolic signatures may not explain the link between lifestyle and colon cancer, they may still be informative in capturing unique metabolic profiles of the different lifestyle behaviours. Although several features contributing to the HLI metabolic signature were also present in signatures for BMI, alcohol, and smoking, the metabolic profiles of individual lifestyle components appeared distinct, with minimal feature overlap between signatures, suggesting that each lifestyle behaviour may impact metabolic health through different mechanisms. Biological interpretation of the metabolites, however, requires annotation of the untargeted metabolic features, and given that neither the metabolic signatures nor the individual features displayed associations with the outcome, the investment of the necessary time and resources to perform feature annotation was not justified in this study.

This study had several strengths. It was the first to apply a weighted, data-driven method of calculating the HLI, a method developed to reduce the bias associated with composite scores, to improve the predictive power of the HLI score, and to provide more accurate estimates of the relationship between lifestyle and disease risk [31]. Rather than assuming that each lifestyle component contributed equally to disease risk, using this method, each lifestyle component was weighed based on their associations with the disease outcome. Furthermore, in addition to analysing the adherence to a healthy lifestyle as a whole, this study also included component-specific analyses to better examine the role of each lifestyle factor on the risk of colon cancer.

However, this study also had some limitations. First, this study relied on untargeted metabolic data acquired in blood samples collected at recruitment, the same time as lifestyle exposure assessment. We assumed that lifestyle behaviours drove changes in metabolic profiles; however, this temporal relationship cannot be definitively established in our study due to the simultaneous assessment of lifestyle data and blood collection. Recent evidence suggests a potential bidirectional relationship where poor metabolic health could contribute to the development of obesity, as well as vice versa [38, 41, 42]. This dual causality complicates the interpretation of our findings and underscores the need for longitudinal studies with repeated measures to clarify the directionality of these associations. Additionally, our analysis on SEP may have been limited by using the highest education level attained to measure SEP. While education is a well-established proxy for SEP in epidemiological studies, other indicators, such as occupation and income, as well as parental education, may provide information to more accurately characterise participants' SEP and investigate its complex interplay with lifestyle factors and disease risk [11, 43, 44].

## Conclusion

Healthy lifestyle behaviours and their serum metabolic signatures were individually inversely associated with colon cancer risk, with stronger associations in men than women and no differences across SEP. The associations between HLI and colon cancer remained after adjustment for the metabolic signature, while the associations between the signature and colon cancer disappeared after accounting for the HLI, suggesting that the mechanisms by which lifestyle impact colon cancer are not fully captured by serum metabolic signatures.

## Supplementary Information

Supplementary Material 1.

## Data Availability

For information on how to request access to EPIC data and/or biospecimens, please refer to https://epic.iarc.fr/access/ for the EPIC-Europe Access Policy.

## Abbreviations

BMI: Body Mass Index
CI: Confidence Interval
EPIC: European Prospective Investigation into Cancer and Nutrition
HLI: Healthy Lifestyle Index
ICD: International Classification of Disease
IQR: Interquartile Range
KNN: K-Nearest Neighbour
LASSO: Least Absolute Shrinkage and Selection Operator
MET: Metabolic Equivalent of Task
MetSig: Metabolic Signature
mrMDS: Modified Relative Mediterranean Diet Score
OR: Odds Ratio
PA: Physical Activity
PC-PR2: Principal Component Partial R-square
SD: Standard Deviation
SEP: Socioeconomic Position
SERRF: Systematic Error Removal using Random Forest
TE: Total Effect
WCRF: World Cancer Research Fund
