# Supplementary material for: Can serum metabolic signatures inform on the relationship between healthy lifestyle and colon cancer risk?
Source: Cancer Metab. 2025 Jun 16;13:30. doi: 10.1186/s40170-025-00388-0 (PMC12168339; doi:10.1186/s40170-025-00388-0)
Supplement: Supplementary file 1 — Supplementary Material 1. [file 40170_2025_388_MOESM1_ESM.docx]

Supplementary Materials Table of Contents

[**Supplementary Methods** 2](#_Toc188612856)

[**Supplemental Tables and Figures** 4](#_Toc188612857)

**Figure S1**. Pre-processing pipeline for untargeted metabolomic data.

**Figure S2**. Pearson correlation of features contributing most to the overall metabolic signature of HLI, sorted by retention time.

**Table S1**. Penalised regression performances for metabolic signature derivation from nested 10-fold cross validation amongst controls. Model performance aims to maximise correlation with the Healthy Lifestyle Index (HLI) while minimising mean squared error (MSE).

**Table S2**. Partial Pearson Correlations between metabolic signatures comparing global metabolic signature of the Healthy Lifestyle Index (MetSig HLI) with subgroup-specific metabolic signatures of HLI, adjusted for sex and socioeconomic position

**Table S3**. Weights of each lifestyle component’s contribution to the overall outcome-specific Healthy Lifestyle Index (HLI) by level of exposure.

**Table S4**. Participant characteristics of the Colon Cancer Nested Case-Control study participants in EPIC by sex and socioeconomic position (SEP).

**Table S5.** ORs (95% CI) for colon cancer risk and lifestyle components or metabolic signatures for each HLI component. All estimates are reported for a 1-standard deviation increase, and all models were stratified by match case-set.

# Supplementary Methods

Methods for sample analysis and raw data pre-processing

**Sample preparation**

The 2,242 samples were thawed at room temperature and prepared by mixing 30 µL of plasma with 200 µL of cold acetonitrile and filtering the precipitate with 0.2 µm Captiva ND plates (Agilent Technologies). A 100-µL aliquot of the filtrate was then mixed with equal volume of ultrapure water in a well plate that was then sealed (Rapid EPS, BioChromato, Fujisawa, Japan), frozen at -80 °C, and thawed at room temperature before analysis. Quality control (QC) samples were prepared from a serum pool that was made by mixing 10-µL aliquots of 400 randomly selected study samples and processed together with the study samples. Blank samples were also prepared along the serum samples in an identical manner, only leaving the serum out of the process. Each well plate included four individually prepared QCs and two blanks.

**Sample analysis**

Samples were analysed as five independent analytical batches, each consisting of five 96-well plates. The samples were randomized across the batches, with the matched case-control pairs analysed subsequently, randomly altering their order of analysis. A QC sample was injected after every ca. 10 samples. Analysis was performed with a UHPLC-QTOF-MS system that consisted of a 1290 Binary LC and a 6550 QTOF mass spectrometer equipped with Jet Stream electrospray ionization source (Agilent Technologies). Samples were kept at 4 °C and 2 µL was injected. An ACQUITY UHPLC HSS T3 column (2.1 × 100mm, 1.8 μm) was maintained at 45 °C and the mobile phase consisted of ultrapure water and LC-MS grade methanol, both with 0.05 % (v/v) of formic acid. Following gradient profile was used: 0–6 min: 5% to 100% methanol, 6–10.5 min: 100% methanol, 10.5–13 min: 5% methanol. Mobile phase flow rate was 0.4 ml/min.

Mass spectrometer drying gas temperature was 175°C and flow 12 L/min, with capillary, nozzle, and fragmentor voltages of 3500 V, 300 V, and 175 V, respectively. The sheath gas temperature was 350°C and flow 11 L/min, and nebulizer pressure 45 psi. Continuous mass axis calibration was employed using lock mass ions *m/z* 121.0509 and 922.0098 in the positive ionization mode and *m/z* 112.9856 and 966.0007 in the negative ionization mode. Data was acquired in centroid format using an extended dynamic range mode, and acquisition rate of 1.67 Hz, over the mass range of 50-1200 Da (MassHunter Acquisition 10.1, Agilent Technologies).

**Data pre-processing**

Pre-processing was performed using Profinder 10.0.2.162 and Mass Profiler Professional B.14.9.1 software (MPP; Agilent Technologies). A “Batch recursive feature extraction (small molecules)” process was employed for samples and blanks to find [M+H]^+^ and [M-H]^-^ ions, in the positive and negative ionization mode, respectively. Height thresholds of 1500 and 8000 counts for mass and chromatographic peaks were used, respectively, and a minimum quality score of 50 in positive mode and 70 in negative mode. Feature alignment between samples was performed with retention time and mass windows of ±0.03 min and ±(10 ppm + 1 mDa), respectively. A target list for the recursive extraction was created by including features found in at least 5 % of the study samples within each batch. For recursive feature extraction, ±25 ppm width was used for the m/z values to draw chromatographic peaks, with Agile 2 integrator and no smoothing, and the mass calculated as an average from spectra >50% peak heights. No filtering was applied. The five batches were processes separately and the resulting features were exported as a .pfa file and aligned in MPP. Features present in every blank sample were excluded, unless 5-fold greater in average intensity in samples, for each batch separately (missing values excluded from the fold change calculation). The remaining features were exported as a .cef file from each batch, and aligned in MPP (RT window 0.07 min, mass window 15 ppm + 2 mDa). The resulting features were exported as a single .cef file that was used as target in a “Batch targeted feature extraction” process in Profinder for all study samples and QCs, using same settings as above, except for RT matching tolerance that was ±0.05 min. The targeted feature extraction was performed independently for each batch, and all the resulting .cef files were aligned in MPP using settings described above. Features present in at least one sample were exported as a .csv file. Chromatographic peak areas were used as a measurement of intensity.

**Quality control**

The intra- and inter-batch quality of the analysis was evaluated using data from the pooled QC samples. The assessment was based on the overall response stability, evaluated by plotting in chronological order the log2-normalized average response of features found in all QC samples. Moreover, the response variability was assessed by calculating the relative standard deviation of the intensity of known compounds in positive and negative ion mode for all QC samples and the QC samples of each batch.

# Supplemental Figures and Tables

**Figure S1**. Pre-processing pipeline for untargeted metabolomic data.

**
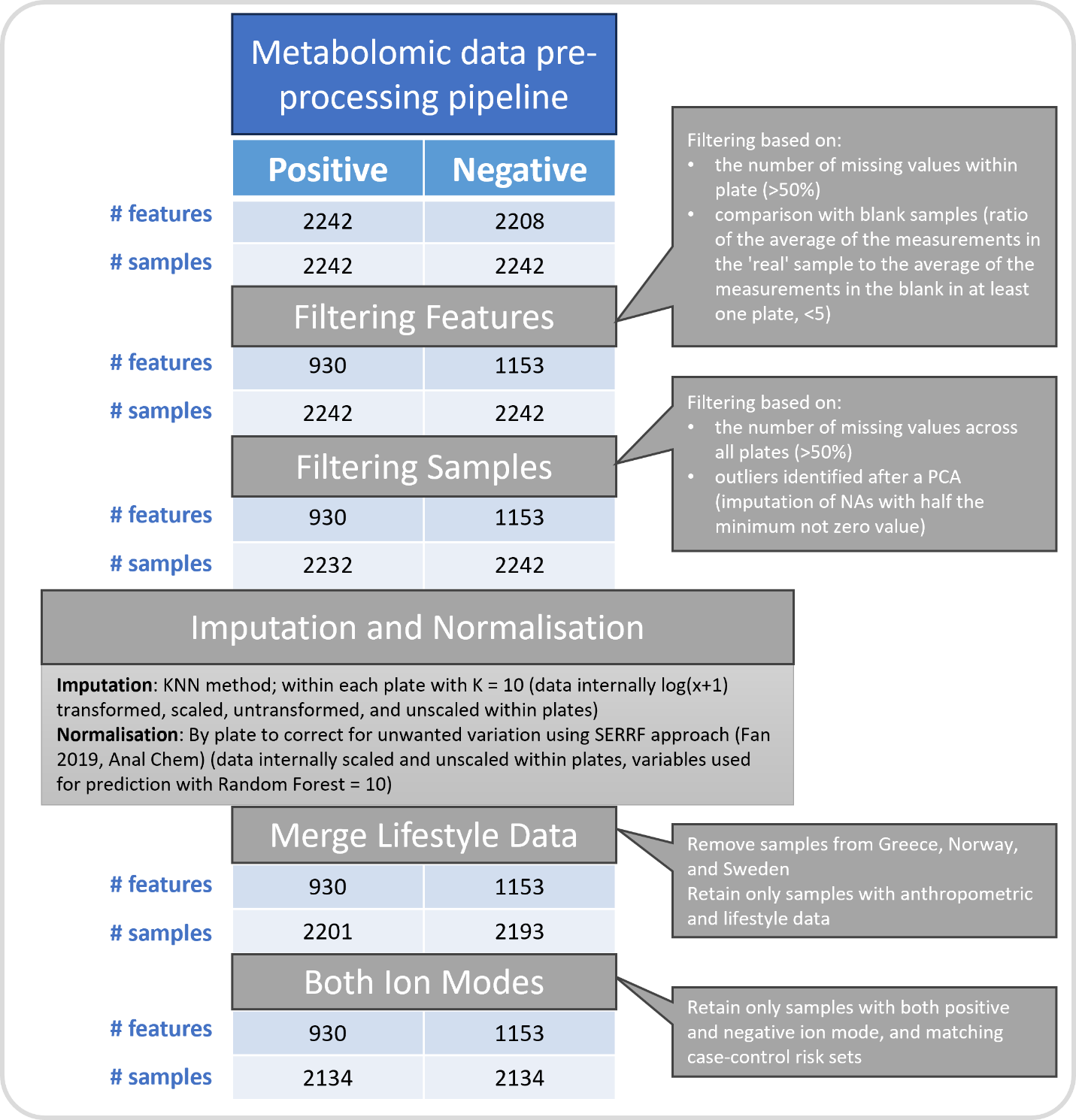
**

**Figure S2**. Pearson correlation of features contributing most to the overall metabolic signature of HLI, sorted by retention time. Feature identities shown as mass@retention time.


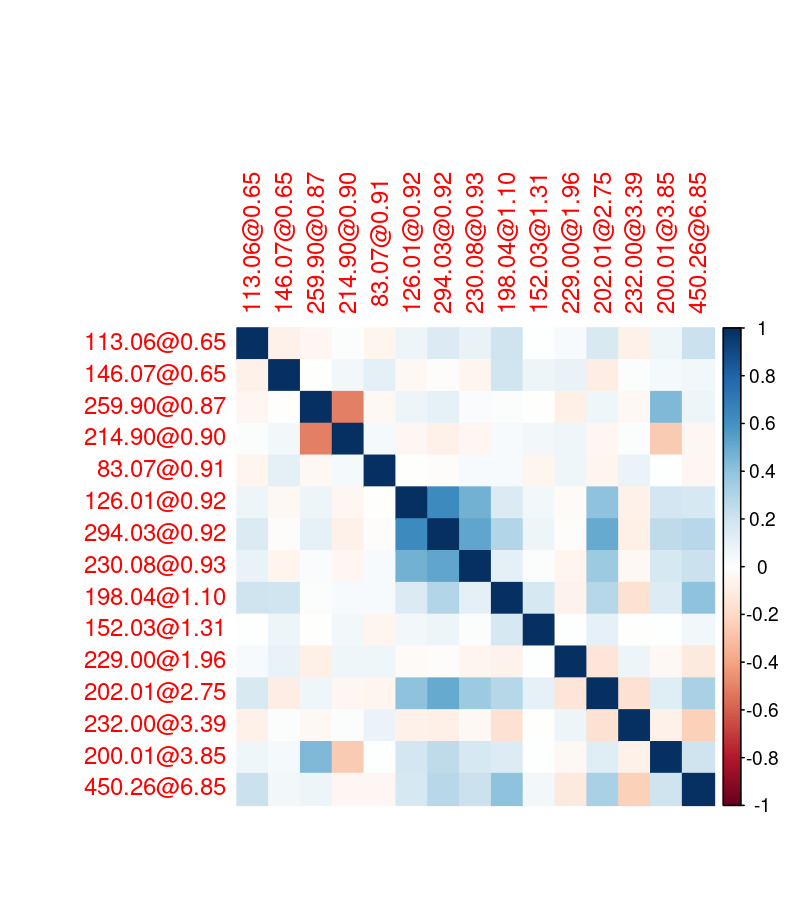


**Table S1**. Penalised regression performances for metabolic signature derivation from nested 10-fold cross validation amongst controls. Model performance aims to maximise correlation with the Healthy Lifestyle Index (HLI) while minimising mean squared error (MSE).

| **Model** | **Package** | **Parameters** | **# Features Retained^1^** | **Correlation^1^ with HLI** | **MSE^1^** |
| --- | --- | --- | --- | --- | --- |
| Partial least squares (PLS) | *Pls* | *ncomp = 1* | 2,083 | 0.42 | 0.83 |
| Sparse PLS | *mixOmics* | *ncomp = 1*  *keepX = c(20, 30, 40, 50, 60, 70, 80, 90, 100, 125, 150, 200)* | 70 | 0.44 | 0.81 |
| LASSO (λmin) | *Glmnet* | *alpha = 1*  *lambda = λmin* | 115 | 0.52 | 0.73 |
| LASSO (λ1se) | *Glmnet* | *alpha = 1*  *lambda = λ1se* | 43 | 0.50 | 0.76 |

^1^Averaged across ten folds

Abbreviations: PLS: partial least squares; LASSO: least absolute shrinkage and selection operator; ncomp = number of components; keepX = number of features to retain in each component; λmin: lambda parameter of minimum mean cross-validated error; λ1se: largest lambda parameter value such that error is within 1 standard error of the cross-validated errors for λmin

**Table S2**. Partial Pearson Correlations between metabolic signatures comparing global metabolic signature of the Healthy Lifestyle Index (MetSig HLI) with subgroup-specific metabolic signatures of HLI, adjusted for sex and socioeconomic position

| **Metabolic Signatures** | **k_feature_** | **Partial Correlations with** | |
| --- | --- | --- | --- |
|  |  | **HLI score** | **MetSig HLI** |
| HLI | 130 | 0.59 (0.56, 0.61) |  |
| Male | 130 | 0.63 (0.59, 0.67) | - |
| Female | 130 | 0.53 (0.49, 0.57) | - |
| Low SEP | 130 | 0.58 (0.54, 0.61) | - |
| High SEP | 130 | 0.60 (0.55, 0.61) | - |
| **Subgroup Specific** |  |  |  |
| HLI Male | 104 | 0.66 (0.63, 0.70) | 0.92 (0.91, 0.93) |
| HLI Female | 58 | 0.52 (0.48, 0.56) | 0.81 (0.79, 0.83) |
| HLI Low SEP | 104 | 0.58 (0.54, 0.61) | 0.95 (0.95, 0.96) |
| HLI High SEP | 46 | 0.57 (0.52, 0.62) | 0.89 (0.97, 0.90) |

Abbreviations: HLI: Healthy Lifestyle Index; MetSig HLI: metabolic signature of the HLI; k_feature_: number of features

**Table S3**. Weights of each lifestyle component’s contribution to the overall outcome-specific Healthy Lifestyle Index (HLI) by level of exposure.

| **Lifestyle component** | **Level of exposure** | **Weight** |
| --- | --- | --- |
| Smoking status | Current > 15 cigarettes/day​ | 0 |
|  | Current ≤15 cigarettes/day​ | -0.24 |
|  | Ex-smokers ≤ 10 years​ | -0.13 |
|  | Ex-smokers > 10 years​ | -0.42 |
|  | Never | -1.20 |
| Alcohol intake [g/day] | ≥ 60​ | 0 |
|  | 24-59.9 | -0.73 |
|  | 12-23.9 | -1.79 |
|  | 6-11.9 | -2.05 |
|  | < 6 | 1.91 |
| BMI​ [kg/m^2^]​ | ≥ 30​ | 0 |
|  | 26-29.9 | -0.36 |
|  | 24.25.9 | -1.17 |
|  | 22-23.9 | -1.31 |
|  | <22 | -1.13 |
| Physical activity [Metabolic Equivalent of Task hours/week] | 1st quintile – Lowest | 0 |
|  | 2nd quintile | -0.24 |
|  | 3rd quintile | -0.38 |
|  | 4th quintile | -0.45 |
|  | 5th quintile – Highest | -0.64 |
| Modified-relative Mediterranean Diet Score [mrMDS] | 1st quintile – Lowest | 0 |
|  | 2nd quintile | -0.14 |
|  | 3rd quintile | -0.62 |
|  | 4th quintile | -0.66 |
|  | 5th quintile – Highest | -0.61 |

**Table S4**. Participant characteristics of the Colon Cancer Nested Case-Control study^1^ participants in EPIC by sex and socioeconomic position (SEP). Unless otherwise indicated, medians (interquartile range [IQR]) are presented for continuous variables and frequencies (%) for categorical variables.

| **Characteristic** | | **Men** | | **Women** | |  | **Low SEP** | | **High SEP** | |
| --- | --- | --- | --- | --- | --- | --- | --- | --- | --- | --- |
|  | | **Controls (N=473)** | **Cases (N=473)** | **Controls (N=594)** | **Cases (N = 594)** |  | **Controls (N=731)** | **Cases (N=718)** | **Controls (N=336)** | **Cases (N=349)** |
| **Sex** | |  |  |  |  |  |  |  |  |  |
| Male | | 473 (100%) | 473 (44.3%) | 0 (0%) | 0 (0%) |  | 324 (44.3%) | 319 (44.4%) | 149 (44.3%) | 154 (44.1%) |
| Female | | 0 (0%) | 0 (0%) | 594 (100%) | 594 (100%) |  | 407 (55.7%) | 399 (55.6%) | 187 (55.7%) | 195 (55.9%) |
| **Age at blood collection [years]** | | 57.1 (51.7, 61.1) | 57.2 (51.8, 61.2) | 57.4 (51.0, 62.2) | 57.5 (51.0, 62.4) |  | 57.4 (51.9, 61.9) | 57.9 (52.1, 62.1) | 56.7 (49.8, 61.2) | 55.8 (50.4, 61.4) |
| **Height [cm]** | | 171 (167, 177) | 172 (168, 178) | 160 (156, 164) | 160 (156, 165) |  | 164 (158, 171) | 165 (159, 172) | 167 (160, 172) | 167 (162, 174) |
| **Weight [kg]** | | 78.5 (71.8, 84.8) | 82.5 (73.5, 90.4) | 65.8 (58.3, 72.8) | 66.5 (59.5, 74.7) |  | 72.0 (63.9, 80.0) | 74.0 (65.5, 85.0) | 69.0 (60.5, 80.5) | 71.0 (62.5, 82.8) |
| **Body Mass Index [kg/m^2^]** | | 26.4 (24.5, 28.7) | 27.6 (25.2, 30.1) | 25.6 (23.0, 28.8) | 25.7 (23.3, 28.9) |  | 26.4 (24.2, 29.2) | 27.3 (24.5, 30.1) | 25.0 (22.7, 27.5) | 25.7 (23.2, 28.3) |
| **Alcohol at recruitment [g/d]** | | 15.1 (4.30, 35.6) | 21.4 (5.07, 40.0) | 3.37 (0.197, 12.4) | 2.30 (0.197, 11.3) |  | 6.70 (0.528, 20.6) | 6.24 (0.432, 24.3) | 9.63 (1.70, 24.9) | 8.15 (1.49, 24.0) |
| **Physical Activity [MET hours/week]** | | 55.5 (33.5, 86.1) | 49.8 (30.5, 82.4) | 101 (67.2, 139) | 98.2 (61.5, 138) |  | 84.0 (48.1, 130) | 81.1 (44.8, 125) | 72.1 (43.4, 107) | 61.6 (39.5, 105) |
| **Modified-relative Mediterranean Diet Score (mrMDS)** | | 28 (23, 33) | 27 (23, 32) | 27 (22, 32) | 27 (23, 32) |  | 27 (22, 32) | 27 (22, 32) | 29 (24, 33) | 27 (23, 32) |
| **Smoking Status** |  | |  |  |  |  |  |  |  |  |
| Current, >15 cigarettes/day | | 50 (10.6%) | 55 (11.6%) | 37 (6.2%) | 45 (7.6%) |  | 66 (9.0%) | 71 (9.9%) | 21 (6.3%) | 29 (8.3%) |
| Current, ≤15 cigarettes/day | | 94 (19.9%) | 101 (21.4%) | 115 (19.4%) | 94 (15.8%) |  | 135 (18.5%) | 129 (18.0%) | 74 (22.0%) | 66 (18.9%) |
| Former, quit ≤10 years | | 63 (13.3%) | 83 (17.5%) | 33 (5.6%) | 50 (8.4%) |  | 57 (7.8%) | 85 (11.8%) | 39 (11.6%) | 48 (13.8%) |
| Former, quit >10 years | | 130 (27.5%) | 117 (24.7%) | 77 (13.0%) | 87 (14.6%) |  | 128 (17.5%) | 123 (17.1%) | 79 (23.5%) | 81 (23.2%) |
| Never | | 136 (28.8%) | 117 (24.7%) | 332 (55.9%) | 318 (53.5%) |  | 345 (47.2%) | 310 (43.2%) | 123 (36.6%) | 125 (35.8%) |
| **Highest Education** | |  |  |  |  |  |  |  |  |  |
| None | | 40 (8.5%) | 44 (9.3%) | 52 (8.8%) | 60 (10.1%) |  | 92 (12.6%) | 104 (14.5%) | 0 (0%) | 0 (0%) |
| Primary | | 167 (35.3%) | 158 (33.4%) | 229 (38.6%) | 190 (32.0%) |  | 396 (54.2%) | 348 (48.5%) | 0 (0%) | 0 (0%) |
| Technical/professional | | 110 (23.3%) | 108 (22.8%) | 109 (18.4%) | 124 (20.9%) |  | 219 (30.0%) | 232 (32.3%) | 0 (0%) | 0 (0%) |
| Secondary | | 56 (11.8%) | 67 (14.2%) | 99 (16.7%) | 109 (18.4%) |  | 0 (0%) | 0 (0%) | 155 (46.1%) | 176 (50.4%) |
| University or higher | | 91 (19.2%) | 83 (17.5%) | 80 (13.5%) | 76 (12.8%) |  | 0 (0%) | 0 (0%) | 171 (50.9%) | 159 (45.6%) |
| Not specified | | 9 (1.9%) | 13 (2.7%) | 25 (4.2%) | 35 (5.9%) |  | 24 (3.3%) | 34 (4.7%) | 10 (3.0%) | 14 (4.0%) |
| **Country** | |  |  |  |  |  |  |  |  |  |
| France | | 0 (0%) | 0 (0%) | 41 (6.9%) | 41 (6.9%) |  | 1 (0.1%) | 4 (0.6%) | 40 (11.9%) | 37 (10.6%) |
| Italy | | 104 (22.0%) | 104 (22.0%) | 179 (30.1%) | 179 (30.1%) |  | 186 (25.4%) | 182 (25.3%) | 97 (28.9%) | 101 (28.9%) |
| Spain | | 147 (31.1%) | 147 (31.1%) | 93 (15.7%) | 93 (15.7%) |  | 191 (26.1%) | 195 (27.2%) | 49 (14.6%) | 45 (12.9%) |
| United Kingdom | | 70 (14.8%) | 70 (14.8%) | 91 (15.3%) | 91 (15.3%) |  | 119 (16.3%) | 107 (14.9%) | 42 (12.5%) | 54 (15.5%) |
| Netherlands | | 20 (4.2%) | 20 (4.2%) | 80 (13.5%) | 80 (13.5%) |  | 61 (8.3%) | 60 (8.4%) | 39 (11.6%) | 40 (11.5%) |
| Germany | | 51 (10.8%) | 51 (10.8%) | 55 (9.3%) | 55 (9.3%) |  | 74 (10.1%) | 74 (10.3%) | 32 (9.5%) | 32 (9.2%) |
| Denmark | | 81 (17.1%) | 81 (17.1%) | 55 (9.3%) | 55 (9.3%) |  | 99 (13.5%) | 96 (13.4%) | 37 (11.0%) | 40 (11.5%) |
| **Tumour site** | |  |  |  |  |  |  |  |  |  |
| Proximal colon | | - | 279 (50.0%) | - | 292 (49.1%) |  | - | 315 (43.9%) | - | 171 (49.0%) |
| Distal colon | | - | 252 (45.2%) | - | 258 (43.4%) |  | - | 354 (49.3%) | - | 156 (44.7%) |
| Colon, unspecified | | - | 27 (4.8%) | - | 44 (7.4%) |  | - | 49 (6.8%) | - | 22 (6.3%) |
| **Healthy Lifestyle Index (HLI)**^2^ | | -0.41 (SD = 1.04) | -0.76 (SD = 1.01) | 0.33 (SD = 0.84) | 0.27 (SD = 0.85) |  | -0.007 (SD = 1.00) | -0.21 (SD = 1.08) | 0.01 (SD = 1.01) | -0.13 (SD = 1.00) |
| **Metabolic Signature of HLI (MetSig HLI)**^2^ | | -0.51 (SD = 1.01) | -0.75 (SD = 0.99) | 0.41 (SD = 0.78) | 0.40 (SD = 0.83) |  | 0.004 (SD = 1.03) | -0.12 (SD = 1.09) | -0.01 (SD = 0.93) | -0.08 (SD = 1.01) |

^1^Matching factors were age, sex, study centre, fasting status, and follow-up time since blood collection. Women were additionally matched for menopausal status and phase of menstrual cycle at blood collection.

^2^Presented as mean (standard deviation [SD])

Abbreviations: SEP: Socioeconomic Position, MET: Metabolic Equivalent of Task

**Table S5.** ORs (95% CI) for colon cancer risk and lifestyle components or metabolic signatures (MetSig) for each HLI component. All estimates are reported for a 1-standard deviation increase, and all models were stratified by match case-set.

| **Lifestyle component** | | **Model 1** | | **Model 2** | | **Model 3** | | **Model 4** | |
| --- | --- | --- | --- | --- | --- | --- | --- | --- | --- |
|  |  | **OR (95% CI)** | **p** | **OR (95% CI)** | **p** | **OR (95% CI)** | **p** | **OR (95% CI)** | **p** |
| Smoking | Smoking | 1.06 (0.97, 1.16) | 0.186 | 1.06 (0.94, 1.19) | 0.350 | 1.08 (0.96, 1.22) | 0.224 | 1.07 (0.95, 1.21) | 0.245 |
|  | Smoking MetSig | 1.04 (0.95, 1.14) | 0.347 | 1.01 (0.89, 1.13) | 0.924 | 0.97 (0.86, 1.10) | 0.679 | 0.97 (0.86, 1.11) | 0.692 |
| Alcohol | Alcohol | 1.09 (0.99, 1.20) | 0.086 | 1.04 (0.93, 1.17) | 0.512 | 1.04 (0.93, 1.17) | 0.486 | 1.04 (0.93, 1.17) | 0.491 |
|  | Alcohol MetSig | **1.11 (1.01, 1.22)** | **0.037** | 1.08 (0.96, 1.22) | 0.179 | 1.07 (0.95, 1.21) | 0.254 | 1.07 (0.95, 1.21) | 0.276 |
| BMI | BMI | **1.24 (1.14, 1.36)** | **<0.001** | **1.18 (1.05, 1.32)** | **0.006** | **1.17 (1.04, 1.32)** | **0.007** | **1.19 (1.06, 1.33)** | **0.004** |
|  | BMI MetSig | **1.20 (1.10, 1.31)** | **<0.001** | 1.09 (0.98, 1.22) | 0.128 | 1.10 (0.98, 1.23) | 0.105 | 1.10 (0.98, 1.23) | 0.096 |
| Physical Activity | Physical Activity | **0.89 (0.81, 0.99)** | 0.031 | **0.90 (0.81, 1.00)** | **0.042** | 0.90 (0.81, 1.00) | 0.057 | 0.91 (0.82, 1.01) | 0.078 |
|  | Physical Activity MetSig | 0.90 (0.79, 1.01) | 0.083 | 0.91 (0.80, 1.02) | 0.115 | 0.95 (0.84, 1.03) | 0.427 | 0.96 (0.84, 1.09) | 0.526 |
| Diet | Diet | 1.00 (0.91, 1.10) | 0.922 | 1.07 (0.96, 1.19) | 0.212 | 1.04 (0.94, 1.16) | 0.434 | 1.04 (0.93, 1.16) | 0.467 |
|  | Diet MetSig | **0.91 (0.83, 0.99)** | 0.029 | **0.88 (0.80, 0.97)** | **0.012** | 0.93 (0.84, 1.03) | 0.167 | 0.93 (0.83, 1.03) | 0.151 |

Model 1. Completed unadjusted model (Cancer ~ Component_i_ and Cancer ~ MetSig Component_i_)
Model 2. Mutually adjusted for Component and component metabolic signature (Cancer ~ MetSig Component_i_ + Component_i_)
Model 3. Adjusted for all lifestyle components (Cancer ~ MetSig Component_i_ + Component_i_ + Component_j_ + Component_k_ + Component_l_ + Component_m_ )
Model 4. Adjusted for all lifestyle components and other covariates (Cancer ~ MetSig Component_i_ + Component_i_ + Component_j_ + Component_k_ + Component_l_ + Component_m_ + Education + Height
